# Biomechanical Investigation of Red Cell Sedimentation Using Blood Shear Stress and Blood Flow Image in a Capillary Chip

**DOI:** 10.3390/mi14081594

**Published:** 2023-08-13

**Authors:** Yang Jun Kang

**Affiliations:** Department of Mechanical Engineering, Chosun University, 309 Pilmun-daero, Dong-gu, Gwangju 61452, Republic of Korea; yjkang2011@chosun.ac.kr; Tel.: +82-62-230-7052; Fax: +82-62-230-7055

**Keywords:** erythrocyte sedimentation rate (ESR), ESR index, aggregation index, blood shear stress, blood flow intensity, capillary chip

## Abstract

Blood image intensity has been used to detect erythrocyte sedimentation rate (ESR). However, it does not give information on the biophysical properties of blood samples under continuous ESR. In this study, to quantify mechanical variations of blood under continuous ESR, blood shear stress and blood image intensity were obtained by analyzing blood flows in the capillary channel. A blood sample is loaded into a driving syringe to demonstrate the proposed method. The blood flow rate is set in a periodic on–off pattern. A blood sample is then supplied into a capillary chip, and microscopic blood images are captured at specific intervals. Blood shear stress is quantified from the interface of the bloodstream in the coflowing channel. *τ*_0_ is defined as the maximum shear stress obtained at the first period. Simultaneously, ESR*_τ_* is then obtained by analyzing temporal variations of blood shear stress for every on period. AI*_I_* is evaluated by analyzing the temporal variation of blood image intensity for every off period. According to the experimental results, a shorter period of *T* = 4 min and no air cavity contributes to the high sensitivity of the two indices (ESR*_τ_* and AI*_I_*). The *τ*_0_ exhibits substantial differences with respect to hematocrits (i.e., 30–50%) as well as diluents. The ESR*_τ_* and AI*_I_* showed a reciprocal relationship with each other. Three suggested properties represented substantial differences for suspended blood samples (i.e., hardened red blood cells, different concentrations of dextran solution, and fibrinogen). In conclusion, the present method can detect variations in blood samples under continuous ESR effectively.

## 1. Introduction

Because red blood cells (RBCs) are more numerous than other cells (such as white blood cells and platelets), the rheological properties of RBCs play a significant role in determining blood flow. Owing to their distinctive advantages (i.e., high surface area/volume and flexible membranes), RBCs are easily deformed under external shear stress [1]. High deformability helps RBCs pass through micron-sized capillaries to transport gases and waste into peripheral tissues [2]. In microcirculation, RBCs are exposed to high shear stress as well as oxidative stress [3]. To resist these stresses and provide sufficient functionality, biochemical and structural alterations of RBCs occur sensitively depending on stress conditions [4]. The mechanical properties of RBCs are then substantially altered. Therefore, significant alterations in the mechanical properties of RBCs can be used as biomarkers for diagnosing such pathological disorders as hypertension [5], diabetes [6,7,8], malaria [9], and cardiovascular diseases [10,11,12]) or detecting storage-induced lesions [13,14,15,16,17,18,19] as well as senescence [4].

Several researchers have reported a substantial correlation between coronary heart disease and the mechanical properties of blood [20,21,22]. Probing the mechanical properties of blood is crucial for diagnosing and monitoring diseases [2,23]. Currently, microfluidic techniques, which have several merits such as a high sensitivity, small volume, short measurement period, and disposability), have been employed extensively to measure the mechanical properties of blood (i.e., viscosity [24,25,26,27], deformability [24,28,29,30,31], hematocrit [32,33], erythrocyte sedimentation rate (ESR) [34,35], and aggregation [36,37,38,39]).

Blood viscosity is often determined by injecting blood into a specific microfluidic device [40]. The blood viscosity obtained at a low shear rate is determined substantially by RBC aggregation. RBC deformation or alignment contributes to decreasing blood viscosity at higher shear rates. In a microfluidic environment, it is extremely difficult to obtain consistent viscosity at low shear rates (or flow rates) because a syringe pump does not supply consistent blood flow at extremely low flow rates [41]. Instead of blood viscosity, RBC aggregation has been obtained by analyzing blood flow at extremely low flow rates or stasis [35,42,43]. After blood is injected into a microfluidic channel, the blood flows or stops with external sources (i.e., a solenoid valve [44], pinch valve [35], vibration motor [45], and pump). Unlike RBC aggregation, the ESR is obtained without external flow controllers. When blood (approximately 1 mL) is loaded into a vertical capillary tube, gravity forces the RBCs to fall to the bottom of the tube. When the ESR in the tube is obtained, RBC aggregation causes an increase in cell-to-cell interactions and large voids in the network, resulting in faster sedimentation. The ESR is then interpreted as a comparable measure of RBC aggregation [34]. The interface movement after 1 h is called the sedimentation velocity (mm/h). The sedimentation velocity is routinely used as an ESR index to detect chronic diseases and immune disorders [3,46]. However, the conventional ESR test does not provide information on variations in RBC distributions resulting from RBC sedimentation. Instead of a vertical capillary tube, a driving syringe is employed to supply blood to the microfluidic device. As RBC sedimentation occurs in the syringe, the hematocrit increases in the bottom regions of the syringe. When supplying blood to a microfluidic channel from the bottom region, variations in the RBCs in the tube can be monitored by analyzing the image intensity of the blood flow. Alterations in image intensity are interpreted as an increase in the hematocrit resulting from RBC sedimentation [47,48,49,50]. However, image intensity does not provide mechanical information about the blood. More recently, our group suggests the shear stress for monitoring the effect of the hematocrit on blood flow because it is influenced by the hematocrit [51]. A new ESR index (ESR*_τ_*) has been suggested using the blood shear stress under continuous blood flow. This index provides consistent results compared with the conventional sedimentation velocity. However, the image intensity of the blood flow remains unchanged under continuous blood flow. It is impossible to compare the two indices obtained using blood shear stress and image intensity. To compare the two indices quantitatively, it is necessary to stop and run the blood flow because RBC aggregation occurs at stasis or extremely low shear rates.

In this study, by supplying or stopping blood flow into a microfluidic device with a syringe pump, the blood shear stress and image intensity were obtained at the turn-on and turn-off periods, respectively. Two indices for quantifying the ESR in the driving syringe were obtained by analyzing the blood shear stress and blood image intensity at specific intervals. The contributions of the blood delivery period, air cavity secured into the syringe, and hematocrit to the performance were quantified by measuring the variations in the two indices. To compare the indices quantitatively, several types of suspended blood were prepared by adding normal RBCs to the diluent (i.e., dextran solution and fibrinogen) and by adding thermally hardened RBCs to the dextran solution.

Compared with the conventional ESR method (i.e., sedimentation velocity), the proposed method can monitor the contribution of continuous ESR in a driving syringe to the mechanical properties of blood flows. The variation in the ESR in the driving syringe is then monitored by temporal variations of blood shear stress and blood image intensity. The maximum shear stress measured at the first period can be used to detect mechanical differences in blood samples (i.e., hematocrit or diluent). The three biomechanical properties (i.e., τ_0_, ESR*_τ_*, and AI*_I_*) are effective in detecting differences in blood samples. In an experiment of less than 10 min, three suggested properties can detect variations of blood samples under continuous ESR effectively.

## 2. Materials and Methods

### 2.1. Microfluidic Device Fabrication and Experimental Setup Preparation

**Figure 1:**
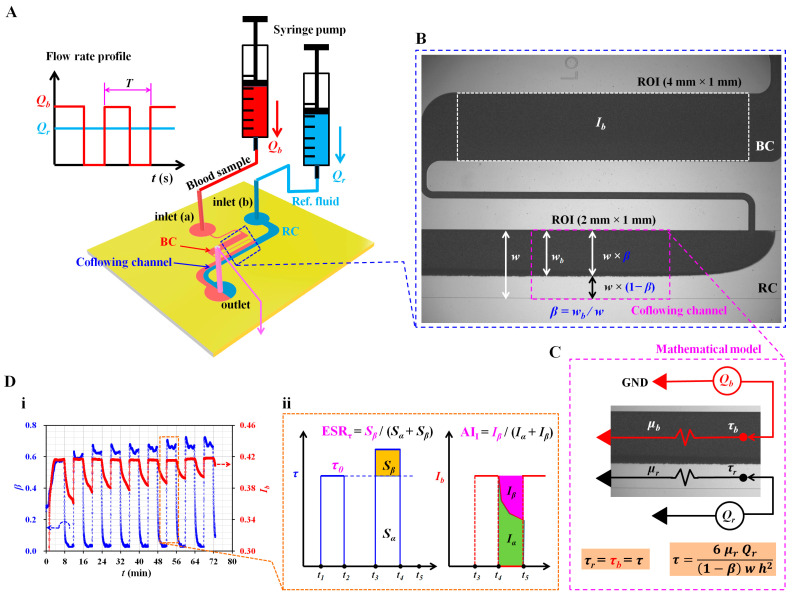
Proposed method for quantification of red blood cell sedimentation using blood shear stress and blood flow image in a capillary chip. (**A**) Schematics of experimental setup, including a microfluidic device and two syringe pumps. The microfluidic device consisted of two inlets (a, b), reference channel (RC), blood channel (BC), coflowing channel, and one outlet. As shown in left side panel, using two syringe pumps, flow rate of reference fluid is set to constant value of *Q_r_*. Flow rate of blood (*Q_b_*) set to square wave profile (i.e., amplitude, period [*T*]). (**B**) Quantification of image intensity and shear stress of microscopic blood flow. Selection of two regions of interest (ROI_S_) for estimating image intensity (*I_b_*) in the blood channel and interface (*β*) in coflowing channel. (**C**) Estimation of blood shear stress (*τ*) using discrete fluidic circuit model. (**D**) Quantification of ESR index and aggregation index. (**i**) Temporal variations of *β* and *I_b_* under on-off blood flow (**ii**) Definition of ESR index (ESR*_τ_*) and aggregation index (AI*_I_*) in terms of blood shear stress and blood flow image intensity, respectively.

As shown in Figure 1A, a microfluidic platform was prepared to quantify the continuous ESR occurring in the driving syringe. It consisted of a microfluidic device, two syringe pumps, and an image acquisition system. The microfluidic device was composed of two inlets (a, b), a reference fluid channel (RC, width = 1 mm), a blood channel (BC, width = 1 mm), a coflowing channel (width (*w*) = 1 mm), and one outlet. To stop the blood flow shortly and avoid the invasion of the reference fluid into the blood channel, a relatively small-sized microfluidic channel (width = 100 µm) was positioned between the blood channel and the coflowing channel. The channel depth (*h*) was set to 50 μm.

A polydimethylsiloxane (Sylgard 184, Dow Corning, Midland, MI, USA) microfluidic device was fabricated using a conventional micro-electromechanical system technique and soft lithography [52].

The microfluidic device was positioned on an inverted microscope (IX81, Olympus, Tokyo, Japan) with a 4× objective lens (numerical aperture = 0.1). Two Tygon Tubing (inside diameter = 250 µm, outside diameter = 750 µm, length = 400 mm, Cole-Parmer, Vernon Hills, IL, USA) was fitted into each inlet. The other Tygon Tubing (inside diameter = 250 µm, outside diameter = 750 µm, length = 200 mm) was fitted to the outlet. To repel air in the microfluidic channels and avoid nonspecific binding of plasma proteins to the inner surfaces, the microfluidic channels were filled with bovine serum albumin (2 mg/mL). This condition was maintained for 10 min. Next, 1× phosphate-buffered saline (PBS) was loaded into the microfluidic channels. Two disposable syringes (approximately 1 mL) were filled with blood and a reference fluid (glycerin solution (30%)), respectively.

As shown in the right panel of Figure 1A, the syringes were installed using two syringe pumps (NeMESYS, Cetoni GmbH, Korbußen, Germany) that were installed vertically against the gravitational direction. As shown in the left panel of Figure 1A, reference fluid was supplied at a constant flow rate (*Q_r_* = 0.5, or 1 mL/h). However, the flow rate of the blood sample (*Q_b_*) was set to a square-wave profile (amplitude = 0.5 mL/h, period: *T*).

A high-speed camera (FASTCAM MINI, Photron, Tokyo, Japan) was used to capture microscopic images of the blood flow in the blood channel and the interface in the coflowing channel. The camera speed was set to 2000 fps, and microscopic images were then recorded at intervals of 1 s. Experiments were conducted at a constant room temperature of 25 °C.

### 2.2. Quantification of Blood Flow Inside the Capillary Chip

To quantify the ESR occurring in the driving blood syringe, the image intensities in the blood channel and the interface in the coflowing channel were quantified by analyzing the microscopic blood flow images recorded by the high-speed camera.

First, as shown in Figure 1B, to obtain the image intensity of the blood flow, a specific region of interest (ROI) of 4 mm × 1 mm was selected within the blood channel. The image intensity of the blood flow was obtained by conducting digital image processing using MATLAB 2019 (MathWorks, Natick, MA, USA). The average image intensity (*I_b_*) was calculated by averaging the image intensities distributed within the ROI.

Second, to obtain the interface (or blood-filled width) in the coflowing channel, a specific ROI (2 mm × 1 mm) was selected at the downstream position where the interface remained distinctively straight. Based on the intensity threshold algorithm (Otsu’s method) [53], a grayscale image was converted into a binary image. The blood-filled width (*w_b_*) was obtained by averaging the interfaces distributed within the ROI. The interface was calculated as *β* = *w_b_*/*w*. Based on the interface (*β*), the width of each stream was obtained as *w* × *β* for the bloodstream and *w* × (1 − *β*) for the reference fluid stream.

### 2.3. Simple Mathematical Model for Estimating Shear Stress of Blood Flow in the Coflowing Channel

As shown in Figure 1C, a discrete fluidic circuit model was constructed to estimate the blood shear stress in the coflowing channel. The flow rate of each fluid is represented as *Q_r_* for the reference fluid and *Q_b_* for the blood. Both fluid streams in the coflowing channel were modeled as two fluidic resistances connected in parallel. A symbol (◀) indicates zero value of pressure (i.e., *P* = 0), designated as GND (ground condition). As both fluid streams flowed in the straight coflowing channel, they had the same pressure condition (i.e., the pressure of the reference fluid stream equals the pressure of the bloodstream). According to previous studies [25,54,55], the approximation error between the mathematical model and the real physical model contributed substantially to the worsening of accuracy, especially when an interface was relocated near both walls. To compensate for the approximation error, a correction factor (*C_p_*), which can be obtained by conducting experiments or numerical simulations, was proposed. For a rectangular channel with a low aspect ratio (i.e., *h*/*w* = 50/1000), the shear stress formulas for the reference fluid stream and bloodstream were derived as

(1) $$\tau_{r}=\frac{6\;\mu_{r}\;Q_{r}}{\left(1-\beta \right)w\;h^{2}}$$

and

(2) $$\tau_{b}=\frac{6\;\mu_{b}\;Q_{b}}{C_{p}\;\beta \;w\;h^{2}}$$

Here, the subscripts *r* and *b* represent the reference fluid stream and bloodstream, respectively. In addition, *µ_r_* and *µ_b_* are the viscosity of the reference fluid and viscosity of the blood sample, respectively. The viscosity of the reference fluid (glycerin [30%]) was set to *µ_r_* = 3 *C_p_* [56]. Based on the force balance condition (i.e., the pressure-drop-induced force equals the shear-stress-induced force) in a straight channel, both fluid streams have the same shear stress (i.e., *τ_r_* = *τ_b_* = *τ*) [55]. Thus, it was possible to obtain the shear stress of the bloodstream in terms of Equation (1) or Equation (2). During the continuous ESR in the driving syringe, hematocrit of blood flow was changed over time. Hematocrit changes blood viscosity continuously. As shear stress contributed to varying interface, shear rate of blood flow also varied depending on interface. Thus, it was impossible to obtain blood shear stress with Equation (2). However, viscosity of the reference fluid remained unchanged. Based on Equation (1), the blood shear stress could be estimated easily when an interface was available. In this study, Equation (1) was used to estimate blood shear stress.

### 2.4. Blood Sample Preparation

To prepare suspended blood (i.e., normal RBCs in diluent), concentrated RBCs and fresh frozen plasma (FFP) were purchased from the Gwangju–Chonnam Blood Bank (Gwangju, Republic of Korea). They were stored in a refrigerator prior to the experiments. According to the specific washing procedures [52], normal packed RBCs were collected by discarding the buffy coat. In addition, the FFP was defrosted at room temperature. Autologous plasma was obtained by passing FFP into syringe filtration. Several types of suspended blood were prepared by adding normal RBCs to a specific diluent (i.e., 1× PBS, autologous plasma, dextran solution, and fibrinogen) or hardened RBCs to specific concentrations of dextran solution. To quantify the contribution of the hematocrit to the ESR in the driving syringe, suspended blood was prepared by adding normal RBCs to a specific dextran solution (10 mg/mL). To increase RBC aggregation in normal RBCs, normal RBCs were added to five different dextran solutions (*C_dex_* = 10, 20, 40, 60, and 80 mg/mL) prepared by dissolving dextran powder (Leuconostoc spp., MW = 450–650 kDa; Sigma-Aldrich, St. Louis, MO, USA) in 1× PBS. To quantify the contribution of fibrinogen to the ESR, suspended blood was prepared by adding normal RBCs into three different fibrinogen solutions (*C_fib_* = 4, 8, and 12 mg/mL), which were dissolved by adding human plasma fibrinogen (F3879-250 mg, Sigma Aldrich Co., St. Louis, MO, USA) into autologous plasma. To evaluate the effect of the hardened RBCs on the ESR, blood was prepared by adding normal RBCs into 1× PBS. Two different degrees of fixed RBCs were obtained by exposing blood to either 50 °C for 30 min or 50 °C for 60 min inside a convective oven. After the thermal fixation of RBCs, RBCs were collected by conducting a washing procedure and were suspended into a specific diluent.

## 3. Results and Discussion

### 3.1. Definition of ESR Index and Aggregation Index in Terms of Blood Shear Stress and Blood Image Intensity

To investigate the contributions of the ESR to the two parameters (*β*, *I_b_*) over time, suspended blood was prepared by adding normal RBCs to a specific dextran solution (10 mg/mL). As shown in Figure 1A, the flow rate of the reference fluid was set to 0.5 mL/h. Simultaneously, the flow rate of blood was set to a square-wave profile (i.e., amplitude = 0.5 mL/h, *T* = 8 min). Figure 1D(i) shows temporal variations of *β* and *I_b_*. When the syringe pump for suspended blood was turned on, *β* tended to increase gradually over time. When the blood syringe pump was turned off, the RBC aggregation caused *I_b_* to decrease significantly over time. The variation range of *I_b_* decreased continuously with an increase in the period. Based on these results, *I_b_* can be used to estimate RBC aggregation or the ESR, especially under periodic on–off blood flow. According to a previous study, the ESR in the driving syringe causes a change in the image intensity of the blood flow in a microfluidic channel under periodic on–off blood flow [50]. In addition, it contributes to increasing blood shear stress (*τ*), even under a constant blood flow [51]—that is, *I_b_* and *τ* can be employed to quantify variations in the ESR occurring in the driving syringe. According to Equation (1), blood shear stress can be calculated when *β* is specified. In Figure 1D(ii), variations in *τ* and *I_b_* are redrawn simply with respect to a specific time. First, the initial shear stress (*τ*_0_) (i.e., the reference value) is defined as the maximum shear stress during the first period of blood flow (from *t* = *t*_1_ to *t* = *t*_2_). In the next period of blood flow (from *t* = *t*_3_ to *t*_4_), the ESR in the syringe contributes to an increase in the hematocrit (or viscosity) of the blood flow. Thus, blood shear stress increases significantly when compared with *τ*_0_ as the reference value. Based on an experimental investigation, to calculate the variation range of blood shear stress for a single period, two parameters (*S_α_* and *S_β_*) were suggested and calculated previously [51]:

(3) $$S_{\alpha }=\int_{t_{3}}^{t_{4}}\tau_{0}dt$$

and

(4) $$S_{\beta }=\int_{t_{3}}^{t_{4}}\left(\tau -\tau_{0}\right)dt$$

ESR*_τ_* is then defined as ESR*_τ_* = *S_β_*/(*S_α_* + *S_β_*). If there is little variation in the ESR, the blood shear stress remains unchanged for each period. ESR*_τ_* is then estimated as zero because *S_β_* = 0. As shown in Figure 1D(i), the blood shear stress tends to increase over time—that is, the ESR in the driving syringe caused to increase *S_β_*.

As a comparable parameter to the ESR, the image intensity of blood flow was used to estimate the trends of RBC aggregation in blood. In the absence of blood flow (from *t* = *t*_4_ to *t* = *t*_5_), the RBC aggregation contributed to decreasing *I_b_* over time. Based on previous studies [37,45], two parameters (*I_α_* and *I_β_*) were obtained by analyzing the temporal variations of *I_b_*. AI*_I_* was defined as AI*_I_* = *I_β_*/(*I_α_* + *I_β_*). As shown in Figure 1D(i), the *I_b_* obtained in the absence of blood flow decreased with an increase in the period. Specifically, AI*_I_* tended to decrease with an increase in the period. The ESR can be monitored in terms of AI*_I_*. Subsequently, two indices (ESR*_τ_* and AI*_I_*) were calculated in terms of blood shear stress (i.e., the blood flow condition) and image intensity of blood flow (i.e., the blood flow at stasis), respectively. Initial shear stress and both indices were used to quantify the continuous ESR in the driving syringe.

### 3.2. Contribution of Dynamic Blood Flow to ESR Quantification

In previous studies [35,45], RBC aggregation was obtained by analyzing blood flow after stopping it completely. Under the square-wave profile of blood flow, the blood flow did not stop immediately because of the time delay of the fluidic system (i.e., the time constant) [57]. The time constant was determined by the fluidic resistance and system compliance (i.e., time constant = fluidic resistance × system compliance). Here, two vital factors (the on–off period [*T*] and air cavity secured in the driving syringe (*V_air_*)) were adjusted to investigate the contributions of the time constant to the ESR*_τ_* and AI*_I_*.

**Figure 2:**
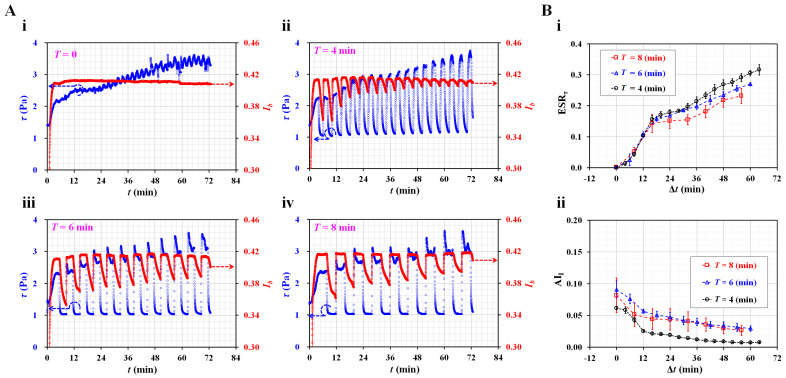
Contributions of on–off period of blood flow (*T*) to ESR index (ESR*_τ_*) and aggregation index (AI*_I_*). (**A**) Temporal variations of blood shear stress (*τ*) and image intensity (*I_b_*) with respect to period (*T*) ((**i**) *T* = 0, (**ii**) *T* = 4 min, (**iii**) *T* = 6 min, and (**iv**) *T* = 8 min). (**B**) Contribution of period (*T*) to ESR*_τ_* and AI*_I_*. (**i**) Temporal variations of ESR*_τ_* with respect to *T* = 4, 6, and 8 min. (**ii**) Temporal variations of AI*_I_* with respect to *T*.

First, to evaluate the contributions of the period of the on–off blood flow to both indices, the period was adjusted from *T* = 0 (continuous blood flow) to *T* = 8 min. The amplitude of the square-wave profile of the blood flow was set to 0.5 mL/h. The flow rate of the reference fluid was set to 0.5 mL/h. To stimulate test blood with a high degree of aggregation, normal RBCs (Hct = 50%) were added to a specific concentration of dextran solution (10 mg/mL) rather than autologous plasma. Figure 2A shows temporal variations of *τ* and *I_b_* with respect to the period (*T*) ((i) *T* = 0, (ii) *T* = 4 min, (iii) *T* = 6 min, and (iv) *T* = 8 min). At a continuous blood flow (*T* = 0), the ESR during driving caused an increase in blood shear stress over time. However, the image intensity did not differ substantially over time. In other words, for quantifying the ESR under continuous blood flow, the blood shear stress was much better than the blood image intensity [51,58]. Shin et al. reported the critical shear stress required for RBC aggregation under transient blood flow [59]. However, under periodic on–off blood flow, the shear stress increased gradually with an increase in the period.

As blood was supplied from the bottom region of the driving syringe, the hematocrit of the blood flow tended to increase over time in the microfluidic channel [50]. As expected, RBC aggregation contributed to a significant decrease in the image intensity in the absence of blood flow. The *I_b_* value changed distinctively depending on the period; however, it decreased substantially over time. Based on the temporal variations of *τ* and *I_b_*, two indices, i.e., ESR*_τ_* and AI*_I_*, were obtained for every period. For continuous blood flow (*T* = 0), ESR*_τ_* = 0.274–0.309 and AI*_I_* = 0. As shown in Figure 2B(i), temporal variations of ESR*_τ_* were obtained with respect to *T* = 4, 6, and 8 min. The ESR*_τ_* was represented as mean ± standard deviation (*n* = 3). Below Δ*t* = 15 min, the ESR*_τ_* increased significantly over time. This period did not have a substantial influence on the ESR*_τ_*. After Δ*t* = 24 min, a shorter period (*T* = 4 min) led to a higher value of the ESR*_τ_* compared with a longer period (*T* = 8 min). Previously, Yeom et al. reported that the oscillational motion of the air cavity caused the ESR to increase in the syringe tube [60]. Shin et al. also showed that the critical shear stress tended to increase gradually during four consecutive periods (i.e., *T* = 5 s) [44]. Based on the previous results, it was inferred that a shorter period contributed to the acceleration of the ESR in the driving syringe. Figure 2B(ii) shows the temporal variations in AI*_I_* with respect to *T*. The AI*_I_* is represented as mean ± standard deviation (*n* = 3). With an increase in the period, the AI*_I_* tended to decrease gradually because of the ESR in the driving syringe. As shown in Figure 2A, the *I_b_* decreased significantly over a longer period (*T* = 6 or 8 min). A longer period led to a higher value of AI*_I_* than a shorter period. After Δ*t* = 24 min, the AI*_I_* did not exhibit a substantial difference between *T* = 6 min and *T* = 8 min. In a previous study, a long period with no blood flow had a positive influence on RBC aggregation over consecutive periods of 6 min [61]; that is, a longer period led to an increase in AI*_I_* compared with a shorter period. In the experimental investigations, a shorter period (*T* = 4 min) resulted in a high sensitivity of ESR*_τ_*. To obtain the ESR in the driving syringe, the period of the square-wave profile was set to *T* = 4 min during all following experiments.

**Figure 3:**
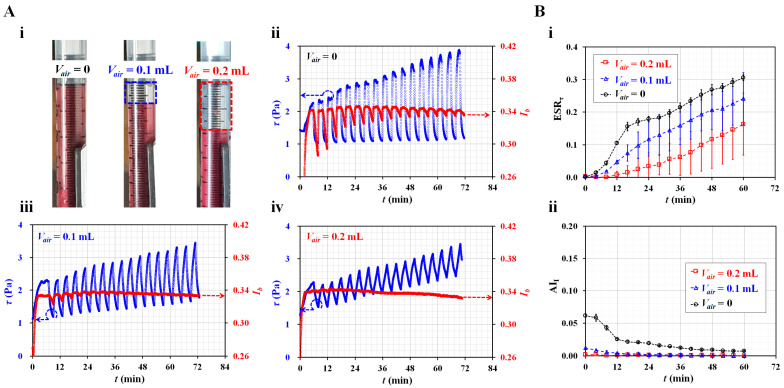
Contribution of air cavity set inside the blood syringe (*V_air_*) to ESR*_τ_* and AI*_I_* in a periodic on-off fashion. (**A**) Temporal variations of blood shear stress and image intensity with respect to air cavity. (**i**) Snapshots for showing air cavity secured inside the driving syringe (*V_air_*) (*V_air_* = 0, 0.1, and 0.2 mL). Temporal variations of *τ* and *I_b_* were obtained with respect to *V_air_* ((**ii**) *V_air_* = 0, (**iii**) *V_air_* = 0.1 mL, and (**iv**) *V_air_* = 0.1 mL). (**B**) Contribution of air cavity to ESR*_τ_* and AI*_I_*. (**i**) Temporal variations of ESR*_τ_* with respect to *V_air_*. (**ii**) Temporal variations of AI*_I_* with respect to *V_air_*.

Second, the compliance effect of the fluidic system caused the time delay to increase or the pulsatile flow to be regulated. Even though the syringe pump was turned off, the blood flow did not stop immediately because of the compliance effect. In the microfluidic system, several components (i.e., a PDMS microfluidic device, flexible tubing, and an air cavity in the syringe) induced compliance effects. Because the RBC aggregation was influenced significantly by dynamic blood flow, it was necessary to quantify the contribution of the compliance effect to the ESR. For convenience, the air cavity secured in the syringe was adjusted to vary the magnitude of the compliance effect. As shown in Figure 3A(i), the air cavity was set to *V_air_* = 0, 0.1, and 0.2 mL by moving a piston in a blood syringe. *V_air_* = 0 indicated that there was no air cavity in the blood syringe. The test blood (Hct = 50%) was prepared by adding normal RBCs to a specific dextran solution (10 mg/mL). The flow rate of the reference fluid was set to 0.5 mL/h. Blood was supplied in an on–off fashion (i.e., amplitude = 0.5 mL/h and *T* = 4 min).

As shown in Figure 3A(ii–iv), temporal variations of *τ* and *I_b_* were obtained with respect to *V_air_*. With respect to *V_air_* = 0.1 and 0.2 mL, blood shear stress increased over time. As the air cavity contributed to a reduction in the alternating components of the blood flow [41,57,62], the fluctuation range of the shear stress decreased significantly at a high air cavity volume. At higher volumes of the air cavity, the blood flow did not stop immediately. Because the air cavity hindered RBC aggregation, the *I_b_* value remained constant over time. Based on temporal variations of *τ* and *I_b_*, two indices (i.e., ESR*_τ_* and AI*_I_*) were obtained over time. Figure 3B(i) shows the temporal variations in ESR*_τ_* with respect to *V_air_*. Here, experiments were repeated twice for each air cavity. The condition without an air cavity (*V_air_* = 0) had a higher value of ESR*_τ_* when compared with *V_air_* = 0.1 or 0.2 mL. Figure 3B(ii) shows the temporal variations of AI*_I_* with respect to *V_air_*. The AI*_I_* had a high value when there was no air cavity. When the air cavity was set to 0.1 or 0.2 mL, AI*_I_* decreased significantly. The experimental results revealed that no air cavity provided a high sensitivity of ESR*_τ_* as well as AI*_I_*. Therefore, to monitor the ESR in the blood syringe with high sensitivity, it was necessary to confirm that any air cavity inside the blood syringe was small.

### 3.3. Contribution of Hematocrit to ESR in Driving Syringe

Previous researchers reported that hematocrit has a strong influence on RBC aggregation [37,39,45,50,54,60,63]. In addition, RBC stiffness contributes to changes in RBC aggregation [38,44,47,52]. As ESR shows similar trends to RBC aggregation, three kinds of experiments were conducted by changing hematocrit, and RBC stiffness. Based on the previous studies, the hematocrit (i.e., RBC volume in relation to total blood volume) was set from 30% to 50%. In addition, to change the stiffness of the RBC, normal RBCs were hardened thermally. To accelerate RBC aggregation, a dextran solution (10 mg/mL) was selected as the diluent. The test blood was prepared by adding normal or hardened RBCs to a specific dextran solution.

The ESR in the syringe caused variations in the hematocrit of the blood flow, which contributed to the shifting of the interface toward the channel wall. When the interface was relocated near the channel wall, the modeling accuracy of Equation (1) deteriorated [55]. Thus, the flow rate of the reference fluid was adjusted from 0.5 to 1 mL/h (i.e., *Q_r_* = 1 mL/h). Test blood was supplied at the square-wave profile (amplitude = 0.5 mL/h, period = 4 min).

**Figure 4:**
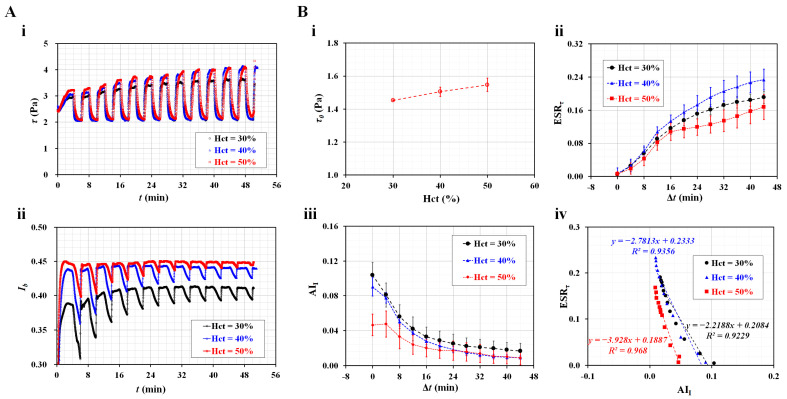
Contribution of hematocrit to ESR index as well as aggregation index. (**A**) Temporal variations of blood shear stress and image intensity with respect to hematocrit. (**i**) Temporal variations of *τ* with respect to Hct = 30%, 40%, and 50%. (**ii**) Temporal variations of *I_b_* with respect to Hct. (**B**) Contribution of hematocrit to initial shear stress (*τ*_0_), ESR*_τ_*, and AI*_I_*. (**i**) Variations of *τ*_0_ with respect to Hct. (**ii**) Temporal variations of ESR*_τ_* with respect to Hct. (**iii**) Temporal variations of AI*_I_* with respect to Hct. (**iv**) Linear relationship between ESR*_τ_* and AI*_I_* with respect to Hct.

First, the contributions of the hematocrit to the ESR were obtained by changing the Hct ranging from 30 to 50%. Figure 4A(i) shows temporal variations of *τ* with respect to Hct. As expected, the hematocrit caused an increase in blood shear stress. The *τ* increased significantly over time. Figure 4A(ii) shows the temporal variations of *I_b_* with respect to Hct. Under blood flow, the hematocrit caused *I_b_* to increase. A significant difference was observed between Hct = 30% and Hct = 50%. Previous researchers reported that RBC aggregation or ESR tended to decrease with increasing hematocrit [50,63,64]. Because the fluctuation range of *I_b_* (i.e., *I_β_*, as shown in Figure 1C(ii)) was proportional to the magnitude of RBC aggregation, it tended to decrease substantially with respect to Hct. In addition, the variation range of *I_b_* decreased gradually over time. As shown in Figure 1C(ii), *τ*_0_ was obtained as the maximum shear stress during the first period. For convenience, the elapsed time was reset to zero (i.e., Δ*t* = 0). As shown in Figure 4B, the variation of the ESR was summarized in terms of three physical properties, i.e., *τ*_0_, ESR*_τ_*, and AI*_I_*. Three representative properties of blood samples are expressed as mean ± standard deviation (*n* = 4). Figure 4B(i) shows variations of *τ*_0_ with respect to Hct. According to Equation (2), the shear stress is proportional to blood viscosity. Because Hct was strongly related to blood viscosity [24,65,66], it was reasonable that the Hct contributed to a substantial increase in *τ*_0_.

Figure 4B(ii) shows temporal variations of ESR*_τ_* with respect to Hct. Interestingly, within 16 min, the ESR*_τ_* did not differ substantially with respect to Hct. Based on the results, the ESR*_τ_* obtained within a short duration (less than 16 min) did not depend on the Hct in the range from 30 to 50%. However, after 20 min, the ESR*_τ_* tended to decrease with respect to the Hct. According to a previous study [51], the ESR*_τ_* decreased at a higher Hct under continuous blood flow. The ESR*_τ_* did not exhibit a substantial difference between Hct = 30% and Hct = 40%, but it did show a substantial difference between Hct = 30% and Hct = 50%. Thus, it was inferred that the ESR*_τ_* tended to decrease at a higher Hct without respect to the blood flow pattern (i.e., continuous or square-wave blood flow). Figure 4B(iii) shows the temporal variations of AI*_I_* with respect to Hct. The AI*_I_* did not exhibit a substantial difference between Hct = 30% and Hct = 40%. A high Hct (50%) caused a decrease in AI*_I_* when compared with a low Hct (30 or 40%). After 16 min, the Hct did not contribute to variations in AI*_I_*. The trends of AI*_I_* were quite similar to those of ESR*_τ_* with respect to the Hct. To find out the relationship between the two indices, the ESR*_τ_* and AI*_I_* (i.e., Figure 4B(ii,iii)) were replotted on the Y-axis and X-axis, respectively. A linear regression analysis was performed using Microsoft Excel Ver. 2019 (Microsoft, Redmond, WA, USA). According to the results, the slope of each Hct was obtained as (a) ΔESR*_τ_*/ΔAI*_I_* = −2.2188 (Hct = 30%), (b) ΔESR*_τ_*/ΔAI*_I_* = −2.7813 (Hct = 40%), and (c) ΔESR*_τ_*/ΔAI*_I_* = −3.928 (Hct = 50%). Both indices had a reciprocal relationship (i.e., negative slope). The slope tended to increase with respect to Hct. Because the regression coefficients had higher values of *R*^2^ = 0.923–0.968, it was confirmed that the indices had a strong linear relationship.

**Figure 5:**
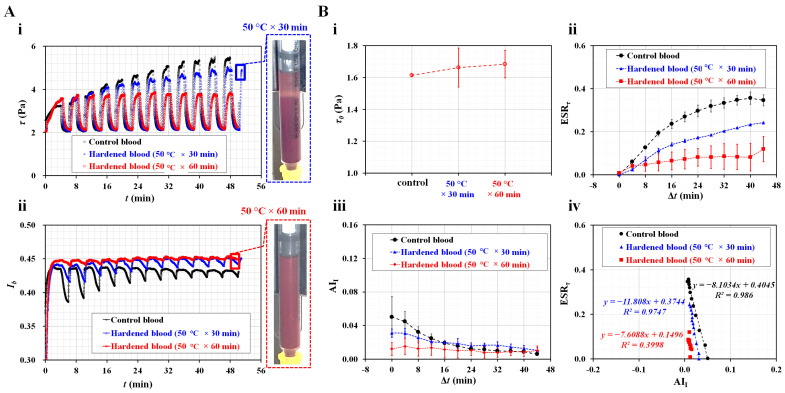
Detection of thermally hardened RBCs in terms of three suggested properties. (**A**) Temporal variations of shear stress and image intensity with respect to control blood and two hardened blood samples. As shown in right side panel, two snapshots of blood syringe filled with heat-treated RBCs (i.e., 50 °C × 30 min and 50 °C × 60 min) were captured at the end of experiment. (**i**) Temporal variations of τ with respect to control blood and two hardened blood samples. (**ii**) Temporal variations of *I_b_* with respect to blood samples. (**B**) Quantification of thermally hardened RBCs in terms of *τ*_0_, ESR*_τ_*, and AI*_I_*. (**i**) Variations of *τ*_0_ with respect to heat treatment conditions. (**ii**) Temporal variations of ESR*_τ_* with respect to heat treatment condition. (**iii**) Temporal variations of AI*_I_* with respect to heat treatment condition. (**iv**) Linear relationship between ESR*_τ_* and AI*_I_* for three blood samples.

Second, two indices were employed to quantify the ESR of the hardened RBCs in the blood syringe. According to previous studies [44,50,67,68], normal RBCs were thermally hardened at high temperatures of either 50 °C for 30 min or 50 °C for 60 min. Hardened blood samples (Hct = 50%) were prepared by adding the hardened RBCs to a specific dextran solution (10 mg/mL). To quantify the contribution of the hardened RBCs to the ESR in the blood syringe, *τ* and *I_b_* were obtained over time. As shown in Figure 5A(i), temporal variations of *τ* were obtained with respect to normal and hardened blood, i.e., 50 °C × 30 min and 50 °C × 60 min. When compared with normal RBCs, thermally shocked RBCs caused the variation range of *τ* to decrease substantially over time. For hardened RBCs, i.e., 50 °C × 60 min, *τ* remained constant over time. The longer the exposure time to thermal shock, the smaller the variation range of the shear stress. Highly hardened RBCs did not contribute to a change in *τ* over time.

Figure 5A(ii) shows the temporal variations of *I_b_* with respect to the control blood and hardened blood. The variation range of *I_b_* tended to decrease with longer heat treatment exposure times—that is, hardened RBCs at 50 °C × 60 min had a smaller *I_b_* range compared with normal RBCs. The right panel shows two snapshots of the blood syringe filled with hardened RBCs with respect to the heat treatment conditions (50 °C × 30 min and 50 °C × 60 min) captured at the end of the experiment. Slightly hardened RBCs (50 °C × 30 min) exhibited a clear interface in the blood syringe. However, there was no clear interface between the diluent and RBCs in the blood syringe for highly hardened RBCs (50 °C × 60 min). According to the results, when normal RBCs were exposed to heat treatment (50 °C × 60 min), the blood shear stress and image intensity did not exhibit substantial differences over time. Furthermore, the ESR was not detected in the blood syringe. As shown in Figure 5B, to quantify the thermally hardened RBCs, three properties (*τ*_0_, ESR*_τ_*, and AI*_I_*) were summarized as mean ± standard deviation (*n* = 2 or 3). Figure 5B(i) shows variations of *τ*_0_ with respect to the heat treatment condition. Although *τ*_0_ tended to increase gradually with respect to the heat treatment exposure time, there was no statistical difference between 30 min and 60 min. Figure 5B(ii) shows temporal variations of ESR*_τ_* with respect to the heat treatment condition. Compared with normal RBCs, the ESR*_τ_* tended to decrease substantially with respect to the heat treatment exposure time. The longer exposure time (60 min) had the smallest ESR*_τ_* variation. Figure 5B(iii) shows the AI*_I_* temporal variations with respect to the heat treatment conditions. The AI*_I_* tended to decrease over time. After 16 min, the AI*_I_* did not change over time. Before Δ*t* = 16 min, the AI*_I_* exhibited a promising difference with respect to the heat treatment conditions. For the normal blood (i.e., no thermally exposed RBCs), continuous ESR occurs in the driving syringe [50]. Hematocrit of blood increases gradually, and it contributes to decreasing AI*_I_*. It was inferred that one hardened blood (i.e., 50 °C for 30 min) contributed to continuous ESR in the driving syringe to a certain degree. The AI*_I_* was then decreased over time. However, the other hardened blood (i.e., 50 °C for 60 min) did not contribute to ESR in the driving syringe. For significantly hardened blood, hematocrit remained constant over time. For this reason, highly hardened RBCs (50 °C × 60 min) did not exhibit a substantial difference in AI*_I_* over time. As shown in Figure 5B(iv), an X–Y plot (X-axis: AI*_I_*, Y-axis: ESR*_τ_*) was constructed to validate the correlation between the two indices. Because the highly hardened RBCs (50 °C × 60 min) did not show a substantial variation of ESR*_τ_* and AI*_I_*, the indices did not have a linear relationship (i.e., *R*^2^ = 0.4). However, with respect to the control blood and slightly hardened blood (50 °C × 30 min), the coefficients of linear regression were estimated to have high values of *R*^2^ = 0.974–0.986. Thus, the two indices had a strong linear relationship. The experimental investigations indicated that the two indices could be used to detect differences between normal and hardened RBCs with sufficient consistency.

### 3.4. Contributions of Diluent to ESR in the Driving Syringe

According to previous studies, diluents (dextran solution [45,51,52,63,69,70,71,72,73] and fibrinogen [34,74]) contribute to increasing RBC aggregation or ESR [75]. To quantify the effect of the diluent on the ESR, test blood was prepared by adding normal RBCs to two types of diluent (dextran solution and fibrinogen). The present method was used to measure variations of the two indices for the test blood.

**Figure 6:**
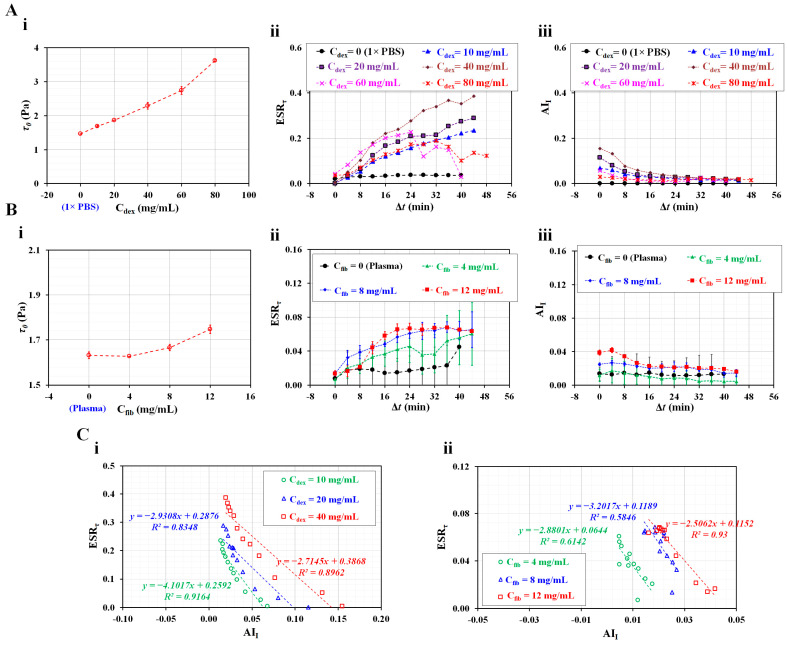
Detection of different diluents (i.e., dextran solution, fibrinogen) in terms of *τ*_0_, ESR*_τ_*, and AI*_I_*. (**A**) Detection of difference in dextran concentration in terms of three properties. (**i**) Variations of *τ*_0_ with respect to *C_dex_* = 0, 10, 20, 40, 60, and 80 mg/mL. (**ii**) Temporal variations of ESR*_τ_* with respect to *C_dex_*. (**iii**) Temporal variations of AI*_I_* with respect to *C_dex_*. (**B**) Detection of difference in fibrinogen concentration in terms of three properties. (**i**) Variations of *τ*_0_ with respect to *C_fib_* = 0, 4, 8, and 12 mg/mL. (**ii**) Temporal variations of ESR*_τ_* with respect to *C_fib_*. (**iii**) variations of AI*_I_* with respect to *C_fib_*. (**C**) Correlation between ESR index and aggregation index for test blood samples diluted by dextran or fibrinogen. (**i**) Linear relationship between ESR*_τ_* and AI*_I_* with respect to concentration of dextran. (**ii**) Linear relationship between ESR*_τ_* and AI*_I_* with respect to concentration of fibrinogen.

First, the proposed method was used to detect variations of two indices for test blood, which was prepared by adding normal RBCs into dextran solution (*C_dex_* = 0, 10, 20, 40, 60, and 80 mg/mL). Here, *C_dex_* = 0 represents 1× PBS. Figure 6A(i) shows variations of *τ*_0_ with respect to *C_dex_*_._ The *τ*_0_ tended to increase significantly with respect to *C_dex_*. Previously, by supplying suspended blood to a microfluidic device under constant blood flow, the shear stress and blood viscosity were obtained with respect to dextran solutions ranging from 5 to 80 mg/mL [51]. In a previous study, it was found that blood viscosity and shear stress tended to increase substantially at higher concentrations of dextran solution, especially under a constant blood flow. However, in the present study, the test blood was supplied to the microfluidic channel in a square-wave profile, as shown in Figure 1A. Because the test blood was prepared with the same normal RBCs, the difference in diluent (i.e., different concentrations of dextran solution) led to an increase in *τ*_0_. Based on Equation (2), it was confirmed that the *τ*_0_ increased because of the higher concentration of the dextran solution. Compared with a previous study, the *τ*_0_ tended to increase substantially, regardless of the blood flow pattern (constant or square wave). The *τ*_0_ could then be used to detect the change in the diluent of test blood. Figure 6A(ii) shows the temporal variations of ESR*_τ_* with respect to *C_dex_*. Below *C_dex_* = 40 mg/mL, the ESR*_τ_* tended to increase substantially with respect to *C_dex_*, which gradually increases during this period. However, above *C_dex_* = 40 mg/mL, the ESR*_τ_* tended to decrease with respect to *C_dex_*. Interestingly, for dextran solutions with *C_dex_* = 60 or 80 mg/mL, the ESR*_τ_* tended to increase for up to 24 min. After 32 min, the ESR*_τ_* tended to decrease over time. According to a previous study conducted under a constant blood flow [51], ESR*_τ_* did not exhibit a substantial difference between 15 and 40 mg/mL. In addition, it significantly decreased between 60 and 80 mg/mL. However, according to the present study, which was conducted at a square-wave blood flow, ESR*_τ_* tended to increase significantly between 10 and 40 mg/mL. Thus, it was inferred that the difference in blood flow pattern (constant or square-wave profile) could lead to different ESR*_τ_* trends. Figure 6A(iii) shows temporal variations of AI*_I_* with respect to *C_dex_*. The AI*_I_* tended to increase up to *C_dex_* = 40 mg/mL. It tended to decrease above *C_dex_* = 40 mg/mL and tended to decrease significantly over time. The results confirmed that the two indices have promise for detecting test blood with different concentrations of dextran.

Second, the present method was employed to detect differences in two indices for test blood, which was prepared by adding normal RBCs into fibrinogen. For healthy control blood, fibrinogen levels ranging from 2 to 4 mg/mL were considered to be the normal range [69]. According to an optical tweezer study, disaggregating force increased significantly above *C_fib_* = 4 mg/mL (i.e., the abnormal range). Furthermore, the AI*_I_* increased substantially in the abnormal range [76]. Based on previous studies, the fibrinogen was set to more than 4 mg/mL (i.e., *C_fib_* = 0, 4, 8, and 12 mg/mL). Here, *C_fib_* = 0 means autologous plasma. Figure 6B(i) shows variations of *τ*_0_ with respect to *C_fib_*. The *τ*_0_ remained unchanged below *C_fib_* = 4 mg/mL (i.e., *τ*_0_ = 1.628–1.632 Pa). Above *C_fib_* = 4 mg/mL, the *τ*_0_ of each fibrinogen increased as *τ*_0_ = 1.666 ± 0.014 Pa (*C_fib_* = 8 mg/mL) and *τ*_0_ = 1.747 ± 0.019 Pa (*C_fib_* = 12 mg/mL). The *τ*_0_ increased substantially in the abnormal range (more than 4 mg/mL) compared with the normal range (less than 4 mg/mL). Compared with the previous optical tweezer study [69], the *τ*_0_ had a similar trend with respect to fibrinogen. Figure 6B(ii) shows temporal variations of ESR*_τ_* with respect to *C_fib_*. When compared with autologous plasma (*C_fib_* = 0), fibrinogen contributed to an increase in ESR*_τ_*. The ESR*_τ_* tended to increase at higher concentrations of fibrinogen (i.e., *C_fib_* = 4–8 mg/mL). The ESR*_τ_* tended to increase over time. For higher concentrations of fibrinogen (i.e., *C_fib_* = 8 or 12 mg/mL), when Δ*t* was less than 12 min, the ESR*_τ_* of *C_fib_* = 12 mg/mL was smaller than that of *C_fib_* = 8 mg/mL. After 16 min, there was no substantial difference between *C_fib_* = 8 and *C_fib_* = 12 mg/mL. Figure 6B(iii) shows variations of AI*_I_* with respect to *C_fib_*. At Δ*t* = 0, the AI*_I_* tended to increase with respect to *C_fib_*. However, after Δ*t* = 16 min, the AI*_I_* did not show a substantial difference with respect to *C_fib_*—that is, fibrinogen did not contribute substantially to the change in AI*_I_*. Compared with ESR*_τ_* (Figure 6B(ii)), the variation range of AI*_I_* was much smaller. Therefore, it was inferred that measuring the variation in ESR in terms of AI*_I_* is difficult.

Finally, it was necessary to validate the linear relationship between the two indices. As shown in Figure 6C, ESR*_τ_* and AI*_I_* were constructed using an X–Y plot. Figure 6C(i) shows a linear relationship between ESR*_τ_* and AI*_I_* with respect to three types of dextran solution (*C_dex_* = 10, 20, and 40 mg/mL). The slope varied from −4.101 to −2.715. The coefficient of linear regression was estimated to have a high value of R^2^ = 0.835–0.916. Figure 6C(ii) shows a linear relationship between ESR*_τ_* and AI*_I_* with respect to three types of fibrinogen (*C_fib_* = 4, 8, and 12 mg/mL). The slope varied from −3.202 to −2.506. With respect to *C_fib_* = 4 or 8 mg/mL, the coefficient of linear regression obtained had a lower value of R^2^ = 0.584–0.614 because AI*_I_* did not show distinctive trends over time. However, the higher concentration of fibrinogen (*C_fib_* = 12 mg/mL) had a higher value of R^2^ = 0.93. The linear regression analysis revealed that both indices had a strong correlation (i.e., ESR*_τ_* ~ AI*_I_*), especially under periodic on–off blood flow.

## 4. Conclusions

Three physical properties (*τ*_0_, ESR*_τ_*, and AI*_I_*) were proposed to quantify biophysical variations of blood samples during continuous ESR in the driving syringe. According to the experimental results, a shorter period of *T* = 4 min and no air cavity contributed to getting high sensitivity of the two indices (ESR*_τ_* and AI*_I_*). The ESR*_τ_* and AI*_I_* showed a reciprocal relationship with each other. The *τ*_0_ was linearly proportional to the hematocrit. The contribution of hematocrit (30–50%) to ESR*_τ_* was negligible during the short duration of the experiment (less than 16 min). In conclusion, the three biophysical properties exhibited substantial differences in several types of suspended blood (i.e., thermally hardened RBCs, dextran, and fibrinogen). In the near future, this method will be used to detect mechanical differences in blood collected from patients. Furthermore, it is necessary to improve the present method for testing blood in clinical settings or in vivo conditions.
